# Arbuscular Mycorrhizal Fungi and Endophytic Fungi Activate Leaf Antioxidant Defense System of Lane Late Navel Orange

**DOI:** 10.3390/jof8030282

**Published:** 2022-03-10

**Authors:** Qiu-Shuang Li, Ya-Chao Xie, Mohammed Mahabubur Rahman, Abeer Hashem, Elsayed Fathi Abd_Allah, Qiang-Sheng Wu

**Affiliations:** 1 College of Horticulture and Gardening, Yangtze University, Jingzhou 434025, China; liqs101999@163.com; 2 Yichang Citrus Science Institute, Yichang 443005, China; citrusxie@163.com; 3 Department of Horticulture, City Operations, Park and Road Services, Edmonton, AB T6W 1A3, Canada; mahabubtarek76@gmail.com; 4 Botany and Microbiology Department, College of Science, King Saud University, P.O. Box. 2460, Riyadh 11451, Saudi Arabia; habeer@ksu.edu.sa; 5 Plant Production Department, College of Food and Agricultural Sciences, King Saud University, P.O. Box. 2460, Riyadh 11451, Saudi Arabia; eabdallah@ksu.edu.sa

**Keywords:** antioxidant enzymes, citrus, endophytic fungi, mycorrhiza, oxidative stress

## Abstract

Arbuscular mycorrhizal (AM) fungi and endophytic fungi collectively symbiose well with plants and, thus, stimulate plant growth; however, it is not clear whether field inoculation of the fungi enhances the resistance potential of plants, particularly in citrus. In the present study, we inoculated AM fungi (*Acaulospora scrobiculata*, *Diversispora spurca*, and *D*. *versiformis*) and endophytic fungi (*Piriformospora indica*) on an eight-year-old lane late navel orange (*Citrus sinensis* (L.) Osb) trees grafted on *Poncirus trifoliata* in a field, and we analyzed the response of the leaf antioxidant defense system. Approximately 2 years after inoculation, the root fungal colonization rate and soil hyphal length significantly increased. Fungal inoculation significantly increased the activity of leaf antioxidant enzymes, such as superoxide dismutase, peroxidase, catalase, and ascorbate peroxidase, and the content of non-enzymatic antioxidants, such as reduced ascorbic acid and reduced glutathione. As a result, fungi-inoculated plants maintained lower concentrations of hydrogen peroxide and superoxide anion radicals and lower levels of membrane lipid peroxidation (according to malondialdehyde level) in leaves than uninoculated plants. Among them, inoculation of *D*. *spurca* and *A*. *scrobiculata* showed relatively higher effects in enhancing the antioxidant defense system than the other fungi. Furthermore, inoculation of *D*. *spurca* induced expressions of *CsFe-SOD*, *CsMn-SOD*, *CsPOD*, *CsCAT1*, and *CsPRR7*; inoculation of *A*. *scrobiculata* and *D*. *versiformis* induced expressions of *CsCAT1*; *CsCAT1* and *CsPOD* were also induced by inoculation of *P*. *indica*. All four inoculations almost upregulated expressions of *CsFAD6*. AM fungi had superior effects than endophytic fungi (e.g., *P*. *indica*). According to our findings, inoculation with beneficial fungi, specifically mycorrhizal fungus *D*. *spurca*, activated the antioxidant defense system of field citrus trees, thus, having potentially superior resistance in inoculated plants.

## 1. Introduction

Citrus is an important horticultural crop with a long life cycle, as is one of the most widely planted fruit trees in the world [1]. Citrus plants are mainly planted in mountains and hills, and they are extremely susceptible to biotic and abiotic stresses, such as high temperatures, droughts, cold, diseases, and pests, all of which reduce fruit quality and affect the development of the citrus industry [2,3]. The lane late navel orange (*Citrus sinensis* (L.) Osb) is an important cultivar in the middle region of the Three Gorges Reservoir area in China [4]. The cultivar originates from the limb sport of the Washington navel orange in Australia and it was first documented in 1950 [5]. It has good productivity with high-quality fruit and vigorous growth [6]. Fresh fruits of the lane late navel orange are typically harvested from April to June in China, while other fruits are in low supply [7]. However, lane late navel orange trees in the field face various biotic and abiotic stresses [8]; thus, it is necessary to enhance the stress resistance of this variety.

It is well known that arbuscular mycorrhizal (AM) fungi are abundant in citrus rhizosphere [9]. AM fungi in the soil could establish a symbiotic relationship with many terrestrial plants, including citrus [10]. One the most important characteristics of this AM symbiosis is to help the host absorb nutrients and water that cannot be reached by the root, to enhance the resistance of the host plant to abiotic stress [11,12]. The AM symbiosis between AM fungi and host plants enhances the resistance of host plants to drought, salt stress, high temperature, nutrient stress, aluminum toxicity, and waterlogging stress by affecting their antioxidant enzyme defense systems [13,14,15,16]. Zhang et al. [17] observed an induced expression of *superoxide dismutase* (*SOD*) and *catalase* (*CAT*) genes in roots of trifoliate orange (*Poncirus trifoliata* L. Raf.) seedlings under drought stress after inoculated with *Funneliformis mosseae*. In potted *C*. *sinensis* L. cv. Cara cara trees grafted on trifoliate orange, inoculation with *Glomus mosseae* dramatically increased leaf SOD and CAT activity during soil water deficit and rewatering [18]. These results show the important roles of AM fungi in enhancing the stress tolerance of plants by increasing the antioxidant enzyme defense system. However, all of these studies were carried out under potted conditions, and it is not clear whether similar mycorrhizal effects might be found in citrus plants grown in the field.

Exotic AM fungi have been applied experimentally to citrus trees in the field. Wu et al. [19] applied *F*. *mosseae* and three AM fungal mixtures into the rhizosphere of *C*. *reticulata* Blanco var. Ponkan cv. Jinshuigan grafted on the trifoliate orange. They observed that the two AM fungal treatments partially improved fruit quality and nutrient element content, whilst a mixture of AM fungi showed superior effects compared to single *F*. *mosseae*. However, AM fungi cannot be cultured *in vitro*, which seriously limits its application. 

Endophytic fungi widely exist in the healthy tissues of living plants, and some coexisting endophytic fungi have developed a special relationship with their host plants in the long process of plant evolution, thus triggering a positive response from the hosts [20]. *Piriformospora indica*, an endophytic fungus, can be cultured *in vitro* and presents similar AM functions [21,22]. *P*. *indica*-colonized plants exhibited a better plant growth performance and higher stress resistance than uncolonized plants [23,24]. Early studies also revealed that *P*. *indica* regulated antioxidant enzymes or non-enzymatic antioxidant defense system to resist various stresses, to reduce the damage from reactive oxygen species (ROS) [25,26]. 

Since a large number of studies with potted plants have revealed the enhancement of the host antioxidant defense system by both AM fungi and *P*. *indica*, we hypothesized that field inoculation with AM fungi and *P*. *indica* also activated the host antioxidant defense system to enhance its resistance to stress. The purpose of this study was to assess the effect of field inoculation with AM fungi and endophytic fungi on root fungal colonization, membrane lipid peroxidation, antioxidant enzyme activity, non-enzymatic antioxidant concentration, and antioxidant enzyme gene expression of the lane late navel orange trees.

## 2. Materials and Methods

### 2.1. Fungal Inoculums

Three AM fungal strains, including *Acaulospora scrobiculata* Trappe (BGC HK02A), *Diversispora spurca* (C.M. Pfeiff., C. Walker and Bloss) C. Walker and A. Schüßler (BGC SD03A), and *D*. *versiformis* (P. Karst.) Oehl, G.A. Silva and Sieverd (BGC HUN02B), were provided by the Bank of Glomeromycota in China (BGC) (Beijing, China). Among them, *A*. *scrobiculata*, *D.* *spurca*, and *D. versiformis* were isolated and identified from the rhizosphere of *Cercis chinensis* from Hong Kong (China), *Lycopersicon esculentum* from Shouguang (Shandong, China), and *Cynodon dactylon* from Guiyang (Hunan, China), respectively. The identified spores were propagated under the conditions of trapping plants (white clover) for three months. The white clover plants were harvested, the aboveground parts were removed, and both the roots and growth substrates were collected as the mycorrhizal inoculum after natural air drying, where the roots were cut into 0.5-cm-long root segments. The inoculums contained spores (22 spores/g for *A*. *scrobiculata*; 19 spores/g for *D*. *spurca*; and 23 spores/g for *D*. *versiformis*), sporocarps, hyphae, and AM fungi-colonized root segments. 

*P*. *indica* Sav. Verma, Aj. Varma, Rexer, G. Kost, and P. Franken was kindly presented by Professor Zhi-Hong Tian of Yangtze University (Jingzhou, China). The fungus was inoculated into potato dextrose agar (PDA) medium for one week at 30 °C and activated on a new PDA for one week. Then the mycelium was inoculated on potato glucose liquid medium for 7 days under dark conditions, at 30 °C and 150 r/min. The spore suspension was collected and mixed with distilled water in the ratio of 1:20 as the fungal inoculum. The absorbance value was measured at 600 nm with a spectrophotometer, and the calculated concentration was 1.72 × 10^8^ CFU/mL.

### 2.2. Field Inoculation of Fungi

On 10 May 2019, in a citrus orchard planted with equal height terraces, in Xiakou, Xingshan, Yichang, Hubei, China (31°12′ N, 110°79′ E), 8-year-old lane late navel orange (*Citrus sinensis* (L.) Osb) trees grafted on trifoliate orange with the same growth vigor were selected for the fungal inoculations. The AM fungi-inoculated tree received 800 g of AM fungal inoculums, based on Wu et al. [19]. Within 1 m around the tree trunk, two ditches (400 g AM inoculum per ditch) were excavated, and then an 800 g mycorrhizal agent was evenly divided into two parts and applied into the two ditches, respectively. According to the results of Cheng et al. [10], the inoculum of *P*. *indica* was 9.25 g mycelium and 1.5-L spore suspensions of *P*. *indica*, which were evenly applied to the two ditches around the tree trunk. For non-fungal inoculated treatments, 800 g of autoclaved AM inoculums and 1.5-L autoclaved spore suspensions of *P*. *indica* were applied.

### 2.3. Experimental Design

The experiment was a one-factor experimental design consisting of five treatments in a randomized block design: (i) inoculation with *A*. *scrobiculata*; (ii) inoculation with *D*. *spurca*; (iii) inoculation with *D*. *versiformis*; (iv) inoculation with *P*. *indica*; (v) non-inoculation with exogenous fungi. Each treatment had four replicates (2 trees per replicate), with a total of 40 trees.

### 2.4. Sample Collection

Samples were taken about two years after inoculation of the AM fungi and *P*. *indica*. On 17 April 2021, the experiment ended, and the root, leaf, and soil samples were collected. Roots were collected from two ditches previously inoculated with AM fungi and endophytic fungi. In the inoculation ditch, fine roots were collected for analysis of root fungal colonization. The soil attached to the fine roots was gently shaken off and collected for analysis of hyphal length. A number of summer leaves from different directions were collected from each tree, and these leaves were immediately frozen with liquid nitrogen in the field. Samples were transported to the laboratory and stored at −80 °C for the analysis of biochemical variables and related gene expression levels.

### 2.5. Determination of Root Fungal Colonization and Soil Hyphal Length

Root fungal colonization was determined using the protocol proposed by Phillips and Hayman [27]. A segment taken from the middle part of the root was carefully washed, cut into 1-cm long root segments, cleared with 10% KOH solution at 95 °C for 1.5 h, followed by bleaching with 30% H_2_O_2_ solution, acidified with 0.1 mmol/L HCl solution, and stained with 0.05% trypan blue solution. The fungal colonization in roots was observed directly under biological microscopes, and the number of fungal colonization was counted. The root fungal colonization rate was calculated as the percentage of the number of fungi-colonized root segments versus the total number of root segments [28]. 

Hyphal length in the soil was measured by the method described by Bethlenfalvay and Ames [29]. A 0.5 g soil sample was incubated with 6 mL 0.1 mol/L phosphate buffer followed through mixing. The 0.8 mL of the above solution was mixed with 0.4 mL 0.05% trypan blue solution at 70 °C for 20 min, cooled to room temperature, and finally examined under a microscope.

### 2.6. Determination of Leaf Antioxidant Enzyme Activity

The activity of SOD in leaves was measured by the procedure described by Wu [30]. The 0.5 g of fresh leaf samples were homogenized in 5 mL phosphate buffers (pH 7.8) and centrifuged at 10,000× *g* for 20 min. The 1.5 mL mixture, including 50 μL of supernatants, 300 μL 130 mmol/L L-methionine, 300 μL 750 μmol/L nitrotetrazolium blue chloride, 300 mL 100 μmol/L ethylenediaminetetraacetic acid disodium salt, 300 μL 20 μmol/L riboflavin, and 250 μL distilled water, was illuminated at 4000 Lux for 30 min, and the absorbance value was measured at 560 nm. 

The activity of CAT in leaves was measured by the procedure outlined by Yang et al. [31]. The 0.3 g of fresh leaf sample was incubated in 7 mL 0.1 mmol/L phosphate buffer (pH 7.8) at 4 °C and centrifuged for 15 min at 4000× *g*. The supernatant was used as the CAT assay. The mixture of 0.2 mL supernatants, 0.1 mmol/L phosphate buffer (pH 7.8), and distilled water was incubated at 25 °C for 3 min, and then 0.3 mL 0.1 mol/L H_2_O_2_ was added. The absorbance at 240 nm was recorded every 1 min for a total of four times. One unit (U) of CAT activity was defined as a 0.01 decrease in the absorbance at 240 nm in 1 min.

The activity of peroxidase (POD) in leaves was assayed using the method described by Li et al. [32]. The 0.3 g fresh leaf sample was ground into homogenates with 8 mL 0.1 mmol/L phosphate buffers (pH 5.5) and centrifuged for 10 min at 3000× *g*. The mixture of 2.9 mL phosphate buffer (pH 5.5), 1.0 mL H_2_O_2_, 1.0 mL 0.05 mol/L guaiacol, and 0.1 mL supernatants was orderly placed into a 10 mL centrifugal tube, which was immediately incubated at 34 °C for 3 min. The absorbance of the mixture at 470 nm was determined. A change in the absorbance of 0.01 in 1 min was defined as one enzyme unit (U).

The activity of ascorbate peroxidase (APX) in leaves was determined using the method described by Wu [30]. The 1.0 g fresh leaf sample was homogenized with 5 mL 50 mmol/L potassium phosphate buffer containing 0.1 mmol/L ethylenediaminetetraacetic acid disodium salt at 4 °C and centrifuged for 15 min at 12,000× *g*. The 0.1 mL supernatant was mixed with 0.1 mL 6 mmol/L ASC, and 1.7 mL 50 mmol/L potassium phosphate buffer containing 0.1 mmol/L EDTA-Na_2_ and 0.1 mL 0.06% H_2_O_2_. The absorbance at 290 nm was immediately recorded every 40 s for a total of four times. One unit (U) of APX activity was defined as a 0.01 decrease in the absorbance at 290 nm in 1 min.

### 2.7. Determination of Leaf Non-Enzymatic Antioxidant Concentration

Leaf ascorbic acid (ASC) concentrations were determined using the method described by Wu [30]. Briefly, a 1.0 g fresh leaf sample was homogenized with 5 mL 5% trichloroacetic acid and centrifuged at 15,000× *g* for 10 min. After 1 min, the 0.2 mL supernatant was mixed with 0.2 mL 150 mmol/L NaH_2_PO_4_ and 0.2 mL of distilled water. Subsequently, 400 μL of 10% trichloroacetic acid, 400 μL of 44% H_3_PO_4_ solution, 400 μL of 4% 2,2-dipyridine solution, and 200 mL of 3% ferric chloride solution were added, mixed well, and then held in a water bath at 37 °C for 1 h. The absorbance of the mixture was recorded at 390 nm. 

Leaf glutathione (GSH) concentrations were assayed as per the protocol described by Chen and Wang [33]. The 1.0 g fresh leaf sample was homogenized with 5 mL 50 mmol/L potassium phosphate buffer containing 0.1 mmol/L ethylenediaminetetraacetic acid disodium salt at 4 °C and centrifuged for 15 min at 12,000× *g*. The 0.25 mL of supernatant was mixed with 2.6 mL 150 mmol/L monometallic sodium orthophosphate, 0.15 mL 5,5′-dithiobis-(2-nitrobenzoic acid), which was held in a water bath at 30 °C for 5 min. The absorbance of the mixture was recorded at 412 nm. 

### 2.8. Determination of Degree of Membrane Lipid Peroxidation

Malonaldehyde (MDA) was measured following the method described by Sudhakar et al. [34]. Fresh leaf samples (0.20 g) were homogenized with 5 mL 0.1% (*w*/*v*) trichloroacetic acid and centrifuged at 3000× *g* for 10 min. The mixture, including 2 mL of supernatant and 2 mL of 0.67% thiobarbituric acid, was incubated at 100 °C for 30 min and then centrifuged at 3000× *g* for 10 min. The absorbance was recorded at 450, 532, and 600 nm, respectively. MDA concentrations were calculated according to their suggested formula. 

### 2.9. Determination of Leaf ROS Levels

Hydrogen peroxide (H_2_O_2_) concentrations in leaves were determined using the method described by Velikova et al. [35]. The 0.1 g fresh sample was homogenized in 5 mL 0.1% (*w*/*v*) trichloroacetic acid and centrifuged at 12,000× *g* for 15 min. A 4 mL mixture was comprised of 1 mL 10 mmol/L potassium phosphate buffer (pH 7.0), 2 mL 1 mol/L KI, and 1 mL supernatant, and the absorbance of the mixture was recorded at 390 nm. 

Superoxide anion (O_2_^•^^−^) concentrations in leaves were assayed following the protocol outlined by Wang and Luo [36]. Fresh leaf samples (0.15 g) were homogenized with 5 mL 0.1 mol/L phosphate buffer (pH 7.8) and centrifuged at 4000× *g* for 10 min at 4 °C. The 0.5 mL of supernatant was mixed with 0.5 mL 50 mmol/L phosphate buffers (pH 7.8) and 0.1 mL 10 mmol/L hydroxylamine chloride for 1 h at 25 °C. Subsequently, 1 mL 17 mmol/L sulfanilamide and 1 mL 7 mmol/L α-naphthylamine were added to the mixture at 25 °C for 20 min, and the absorbance was recorded at 530 nm. 

### 2.10. Determination of Leaf Antioxidant Enzyme Gene Expressions

Total RNA from leaves of the lane late navel orange was extracted and purified using a TaKaRa MiniBEST plant RNA Kit (9769; Takara, Dalian, China) according to the manufacturer’s agreement. The RNA integrity was detected by 1% agarose gel electrophoresis. The Bio Photometer Plus 6132 ultra-micro spectrophotometer (Eppendorf, Hamburg, Germany) was used for spectrophotometric analysis, and the concentration and purity of extracted RNA were calculated by the A_260_/A_280_ ratio. The RNA was transcribed into cDNA using a PrimeScript™ RT reagent Kit with gDNA Eraser (RR047A; Takara, Dalian, China). Five antioxidant enzyme protein genes (*CsFe-SOD*, *CsMn-SOD*, *CsCu/Zn-SOD*, *CsPOD* and *CsCAT1*), a fatty acid desaturase (FAD) protein gene *CsFAD6*, and a circadian clock gene *CsPRR7* were selected and obtained from the NCBI database (http://www.ncbi.nlm.nih.gov accessed on 14 January 2022) and *Citrus sinensis* genome database (http://citrus.hzau.edu.cn, accessed on 14 January 2022). Primer Premier 5.0 software was used to design gene primer sequences (Table 1). The primers were synthesized by the Shanghai Bioengineering Co., Ltd. (Shanghai, China), and then qRT-PCR was carried out. The reverse transcribed cDNA was used as the template, using the CFX96 real time PCR Detection System (Bio-Rad Laboratories, Hercules, CA, USA) and fluorescent dye method (2 × AceQ qPCR SYBR Green Master Mix, AiDLab, Beijing, China) were amplified by qRT-PCR. Each treatment had three biological replicates, along with three technical replicates/biological replicates. The *β-actin* was used as a reference gene. The 2^−ΔΔCt^ method [37] was used to calculate the relative expression of genes, and the target genes with non-fungi inoculation were used as the control.

### 2.11. Data Analysis

The data were subjected to one-factor analysis of variance (ANOVA) in the SAS software (v 8.1), significant differences between the treatments was compared by the Duncan’s multiple range test at the level of 0.05. Pearson’s correlation coefficients (*r*) between variables were tested by the CORR procedure in the SAS software.

## 3. Results

### 3.1. Changes in Root Fungal Colonization and Soil Hyphal Length

**Figure 1:**
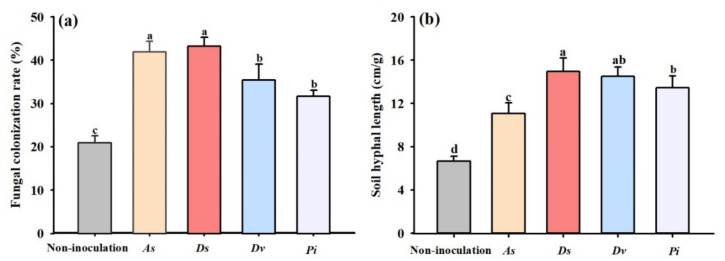
The effect of inoculation with *Acaulospora scrobiculata* (*As*), *Diversispora* *spurca* (*Ds*), *D. versiformis* (*Dv*), and *Piriformospora indica* (*Pi*) on the root fungal colonization rate (**a**) and soil hyphal length (**b**) of the lane late navel orange trees. Data (means ± SD, *n* = 4) are significantly (*p* < 0.05) different if followed by different letters above the bars.

The inoculation of exogenous AM fungi and endophytic fungi significantly increased the root fungal colonization rate of the lane late navel orange trees (Figure 1a). Compared with the non-inoculation, *A. scrobiculata*, *D. spurca*, *D. versiformis*, and *P. indica* significantly increased the root fungal colonization rate by 51.2%, 100.3%, 109.4%, and 69.5%, respectively. The hyphal length in the soil of the lane late navel orange trees also significantly increased (Figure 1b). Compared to the non-inoculation, *A. scrobiculata*, *D. spurca*, *D. versiformis*, and *P. indica* significantly increased the soil hyphal length by 66.3%, 124.4%, 117.5%, and 101.4%, respectively.

### 3.2. Changes in Leaf Antioxidant Enzyme Activity

**Figure 2:**
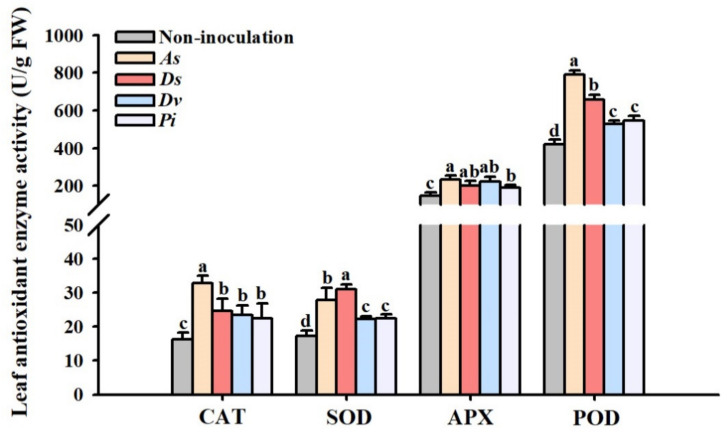
The effect of inoculation with *Acaulospora scrobiculata* (*As*), *Diversispora spurca* (*Ds*), *D*. *versiformis* (*Dv*), and *Piriformospora indica* (*Pi*) on leaf catalase (CAT), superoxide dismutase (SOD), ascorbate peroxidase (APX), and peroxidase (POD) activity of the lane late navel orange trees. Data (means ± SD, *n* = 4) are significantly (*p* < 0.05) different if followed by different letters above the bars.

Fungi-inoculated lane late navel orange trees recorded significantly higher activity of SOD, POD, CAT, and APX in leaves than non-inoculation control: 101.1%, 51.3%, 43.6%, and 37.7% higher CAT activity under *A*. *scrobiculata*, *D*. *spurca*, *D*. *versiformis*, and *P*. *indica*, respectively; 61.5%, 80.4%, 29.1%, and 29.7% higher SOD activity, respectively; 87.6%, 56.4%, 25.4%, and 29.5% higher POD activity and 56.6%, 34.2%, 48.4%, and 26.5% higher APX activity, respectively (Figure 2).

### 3.3. Changes in Sugar Concentrations of Leaves and Roots

**Figure 3:**
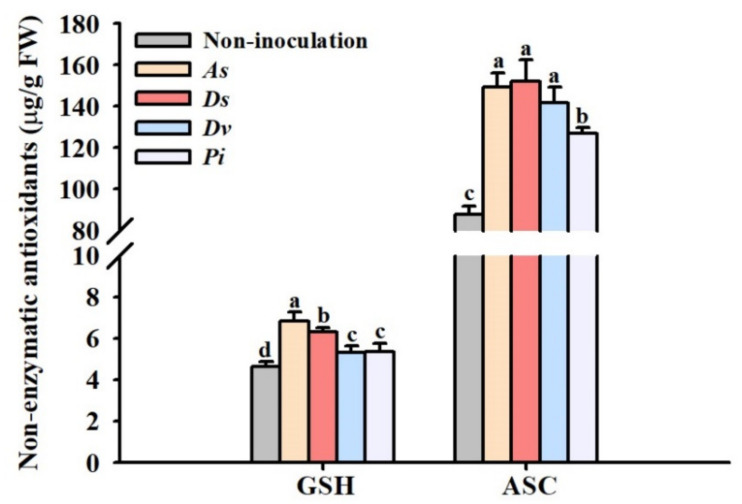
The effect of inoculation with *Acaulospora scrobiculata* (*As*), *Diversispora spurca* (*Ds*), *D*. *versiformis* (*Dv*), and *Piriformospora indica* (*Pi*) on leaf glutathione (GSH) and ascorbic acid (ASC) concentration of the lane late navel orange trees. Data (means ± SD, *n* = 4) are significantly different (*p* < 0.05) if followed by different letters above the bars.

Compared with non-inoculation treatment, inoculation with *A. scrobiculata*, *D. spurca*, *D. versiformis*, and *P*. *indica* significantly increased leaf ASC concentration by 70.2%, 73.5%, 61.9%, and 45.2%, respectively (Figure 3). Similarly, inoculated trees with *A. scrobiculata*, *D. spurca*, *D. versiformis*, and *P. indica* also recorded 46.9%, 36.2%, 14.7%, and 15.3% significantly higher leaf GSH concentration than non-inoculated trees, respectively (Figure 3).

### 3.4. Changes in Expression Levels of Leaf Antioxidant Enzyme Protein Genes

**Figure 4:**
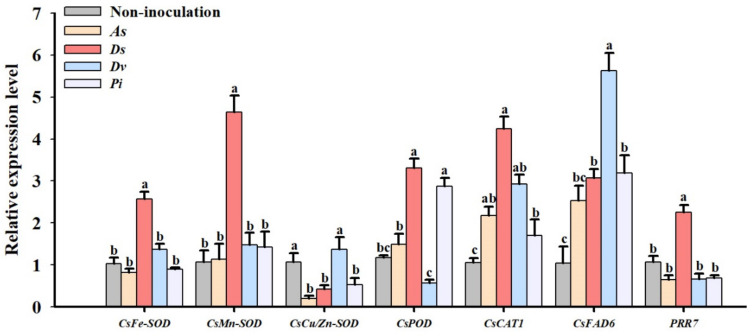
The effect of inoculation with *Acaulospora scrobiculata* (As), *Diversispora spurca* (Ds), *D. versiformis* (*Dv*), and *Piriformospora indica* (*Pi*) on the relative expression level of leaf *CsFe*-*SOD*, *CsMn*-*SOD*, *CsPOD*, *CsCAT1*, *CsFAD6*, and *CsPRR7* in lane late navel orange tree. Data (means ± SD, *n* = 3) are significantly different (*p* < 0.05) if followed by different letters above the bars.

Compared with non-inoculation, inoculation with *D*. *spurca* induced the relative expression level of Cs*Fe-SOD*, Cs*Mn-SOD, CsPOD*, and *CsCAT1* genes by 2.58-fold, 5.64-fold, 3.31-fold, and 4.24-fold, respectively; inoculation of *A. scrobiculata* upregulated the relative expression level of *CsCAT1* by 1.06-fold, but also inhibited the relative expression level of *CsFe-SOD* by 0.64-fold; inoculation of *D. versiformis* activated the relative expression level of *CsCAT1* by 2.93-fold; and inoculation of *P. indica* caused the induced expression of *CsCAT1* and *CsPOD* by 1.71-fold and 2.88-fold, respectively (Figure 4). Furthermore, among the four inoculation treatments, only *D. spurca* induced the expression level of *CsPRR7* up to 2.29-fold, while the other three fungal treatments did not alter the expression of *CsPRR7*. All four treatments almost upregulated the relative expression of *CsFAD6*, which did not differ between non-inoculation and *A. scrobiculata*.

### 3.5. Changes in Leaf ROS Levels

**Figure 5:**
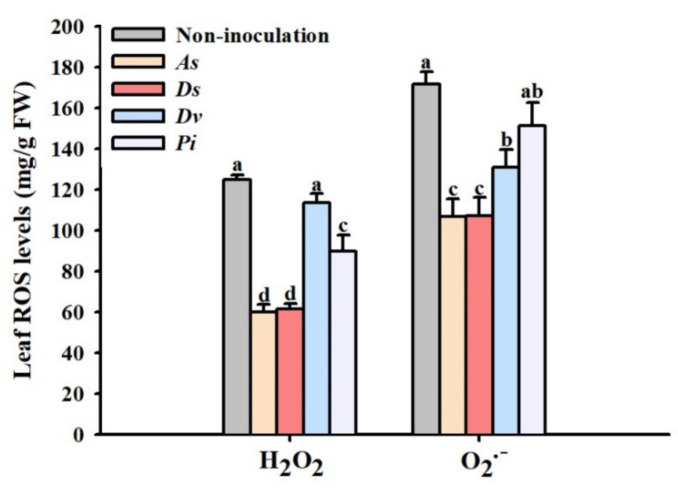
The effect of inoculation with *Acaulospora scrobiculata* (*As*), *Diversispora spurca* (*Ds*), *D*. *versiformis* (*Dv*), and *Piriformospora indica* (*Pi*) on leaf superoxide radical (O_2_^•−^) and hydrogen peroxide (H_2_O_2_) concentrations of lane late navel orange trees. Data (means ± SD, *n* = 4) are significantly different (*p* < 0.05) if followed by different letters above the bars.

Inoculation of *A. scrobiculata*, *D. spurca*, *D. versiformis*, and *P. indica* significantly reduced leaf H_2_O_2_ level by 50.4%, 49.2%, 5.9%, and 25.6%, respectively, as compared with non-inoculation (Figure 5). On the other hand, leaf O_2_^•−^ concentration was significantly decreased by inoculation with *A. scrobiculata*, *D. spurca,* and *D. versiformis* by 28.2%, 28.8%, and 16.2%, respectively, but not *P. indica*.

### 3.6. Changes in Leaf MDA Concentration

**Figure 6:**
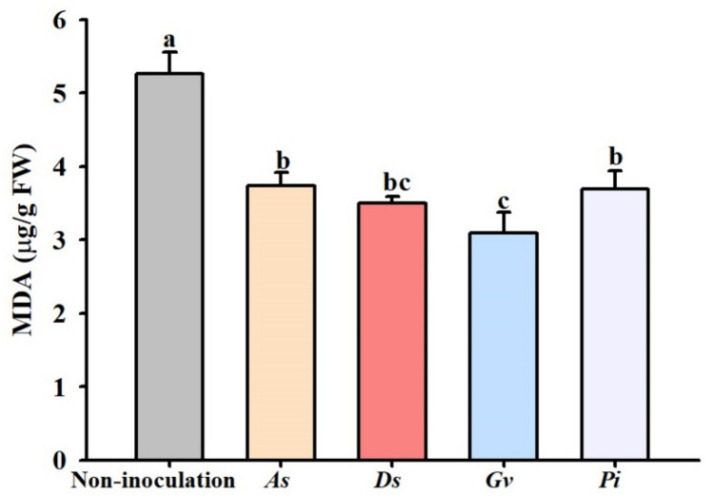
The effect of inoculation with *Acaulospora scrobiculata* (*As*), *Diversispora spurca* (*Ds*), *D*. *versiformis* (*Dv*), and *Piriformospora indica* (*Pi*) on leaf malondialdehyde (MDA) concentrations of lane late navel orange trees. Data (means ± SD, *n* = 4) are significantly different (*p* < 0.05) if followed by different letters above the bars.

Inoculated trees with *A. scrobiculata*, *D. spurca*, *D. versiformis*, and *P. indica* recorded 50.4%, 49.2%, 5.9%, and 25.6% significantly lower leaf MDA concentration, respectively (Figure 6).

## 4. Discussion

For plants with less root hairs, such as citrus, the colonization of AM fungi could efficiently help their roots absorb water and mineral nutrients [38], to improve the adaptability of host plants to stresses [39]. The fungal colonization rate reflects the affinity of fungi with host plants [40]. The water absorption rate of AM fungal hyphae was 2–7 times higher under drought than that of hyphae well-watered, indicating the importance of AM hyphae to plants under drought conditions [39]. The present results showed that non-inoculated trees exhibited root fungal colonization and rich soil hyphae, suggesting that some indigenous AM fungi existed in field citrus rhizosphere. The inoculation of AM fungi and endophytic fungi significantly increased the root fungal colonization rate and soil hyphal length of lane late navel orange trees. Hence, the four fungi could establish a good symbiotic relationship with the roots of lane late navel orange trees in the field, so as to help the host plant tolerate stresses. In *C*. *reticulata* Blanco var. Ponkan cv. Jinshuigan, field inoculation of AM fungi also stimulated root colonization, accompanied by improved fruit quality [19]. At the same time, the colonization by *P*. *indica* also enhanced the resistance of maize to drought [41]. Therefore, it concluded that field-inoculated citrus trees with AM fungi and endophytic fungi had higher root fungal colonization, associated with improved tree resistance and improved fruit quality.

When plants are subjected to stress, it leads to damage in membrane structure, and ROS, such as H_2_O_2_ and O_2_^•−^ accumulates more, causing membrane lipid peroxidation [42,43]. In this study, the inoculated trees with AM fungi and endophytic fungi recorded lower H_2_O_2_ and O_2_^•−^ levels in leaves than uninoculated trees, whilst *A. scrobiculata* and *D. spurca* showed lower ROS levels in leaves than *D. versiformis* and *P. indica*. The result was also observed in leaf MDA content after inoculation with AM fungi and endophytic fungi, indicating less membrane lipid peroxidation occurred in inoculated versus non-inoculated trees. Previous studies also reported lower ROS levels in leaves of *Celtis caucasica* inoculated with *Rhizophagus irregularis* and *F*. *mosseae* under drought and in leaves of cotton inoculated with *P*. *indica* [44,45]. It is known that when AM fungi colonized into roots of the host plant, the efflux of H_2_O_2_ from roots was increased, while the reduced O_2_^•−^ concentration in inoculated plants was associated with the upregulation of antioxidant enzyme gene expression in inoculated plants [46,47].

The production of ROS in plants increases rapidly when they are stressed, coupled with the increase in non-enzymatic antioxidant defense system to maintain metabolic activities of cells [48]. ASC and GSH, as important reducing agents in plants, can directly remove ROS [49]. The present study showed that inoculation of AM fungi and endophytic fungi increased leaf GSH and ASC concentration, among which *A. scrobiculata* and *D. spurca* showed more prominent effects than the other two fungi. A previous study in trifoliate orange also reported that inoculation with *D*. *versiformis* increased ASC and GSH concentration in leaves under ample water and drought stress conditions [50].

In addition, the antioxidant enzyme defense system of stressed plants is also activated simultaneously in response to the oxidative burst to alleviate cellular oxidative damage [51]. SOD converts O_2_^•−^ to H_2_O_2_, which is then converted to H_2_O by CAT or POD. The three antioxidant enzymes thus scavenge ROS of plants and hold them in balance to alleviate cellular oxidative injury [52,53]. APX also assumes an important role in the ASC-GSH cycle [54]. In our study, leaves of lane late navel orange trees inoculated with AM fungi and endophytic fungi recorded significantly higher SOD, POD, CAT, and APX activities, and the inoculation of *A*. *scrobiculata* showed relatively better effects among the four fungal treatments. This is consistent with previous findings in studying the effect of AM fungal inoculation on drought tolerance in the potted trifoliate orange [55]. Furthermore, AM fungal (*Glomus versiforme*) inoculation was able to significantly increase SOD, POD, and CAT activity in tobacco (*Nicotiana tabacum* L. variety Yunyan 87) plants under well-watered and drought conditions, thus reducing oxidative damage [51]. *P*. *indica* was found to improve the salt resistance of tomato, which was related to the increase in SOD, POD, and CAT activity [56]. In fact, *P*. *indica* was isolated from plant roots in the desert environment, having a good potential to resist adversity [21].

Stress significantly affects the expression of stress-responded genes in plants to deal with the stress change [57,58]. Inoculation with AM fungi scavenged ROS accumulated in stressed plants is associated with the regulation of relevant gene expression patterns, to some extent [58]. The present study showed that inoculation with AM fungi and endophytic fungi on lane late navel orange trees regulated the expression level of nine stress-responded genes, dependent on the type of fungi and genes. Among them, inoculation with *D*. *spurca* showed the upregulation of *CsFe*-*SOD*, *CsMn-SOD*, *CsPOD*, and *CsCAT1* expression levels by all fungal inoculations. Such results indicated that *CsCAT1* was a fungi-specific gene, and *D*. *spurca* exhibited a higher antioxidant enzyme defense system in response to stresses. Ding et al. [59] also reported that, at 17:00, *F. mosseae* dramatically upregulated *PtCAT1* expression in the trifoliate orange under well-watered and drought stress conditions. Ye et al. [60] also observed that inoculation with *F. mosseae* alleviated oxidative damage in salt-stressed watermelon through increasing the expression of antioxidant enzyme genes. Hui et al. [57] found that inoculation of *P*. *indica* also upregulated the expression of drought-related protein genes in tobacco plants under drought stress. In our study, *P*. *indica* only induced the expression of *CsPOD* and *CsCAT1*, implying a low capacity of the antioxidant enzyme defense system. In addition, *CsPRR7* expression was increased only in the *D*. *spurca*-inoculated trees, implying that *CsPRR7* was fungi-dependent. Our study also revealed that fungi-inoculated trees recorded higher expression levels of *CsFAD6*, suggesting that inoculated trees trigger more accumulation of unsaturated fatty acids, thus having a strong resistance potential to protect the integrity of the cell membrane [16,61].

## 5. Conclusions

To summarize, inoculation with AM fungi and *P*. *indica* into lane late navel orange trees in fields could increase root fungal colonization and soil hyphal length, along with higher antioxidant enzymatic and non-enzymatic antioxidant defense systems, to reduce ROS accumulation, dependent on fungal species. As a result, inoculated trees recorded high resistance potential during tree growth. AM fungi represented superior effects than endophytic fungi (e.g., *P*. *indica*). Combined with stressed gene expressions and antioxidant defense system responses, *D*. *spurca*, among the four fungi used here, has a high potential value in lane late navel orange trees. Therefore, an AM fungus, *D*. *spurca*, can be appropriately introduced in future cultivation or the nursery stage of lane late navel orange trees to improve root fungal colonization and tree resistance.

## Figures and Tables

**Table 1:** Specific primer sequences of genes used for qRT-PCR.

Gene Name	Gene ID	Primer Sequences (5′→3′)
*CsFe-SOD*	Cs7g19250	F: AGTAAGGAGCGGCGAGTA
		R: GTGGCTAATGCGGTGAAT
*CsMn-SOD*	Cs7g29850	F: GGCGAGCCACCACATAGT
		R: CACCCTCAGCATTCATCTTTT
*CsCu/Zn-SOD*	Cs3g12080	F: GGACCAGCATGGACTACAAGACC
		R: GGATGCCGGTGGAAGTGTTACC
*CsPOD*	Cs1g18600	F: GGCTCAACTTGTCCACCTC
		R: TATCGTCGCCCTGTCTG
*CsCAT1*	Cs3g27280	F: TAACAGTGGAGGAGCGAACA
		R: GGAGCCAGTGCTAAGGGT
*CsFAD6*	Cs8g17450	F: CTGCACGGAGATACAGCTTGGC
		R: GGAATGTGAGGAGCCGTATGATGC
*CsPRR7*	Cs6g03960.1	F: TAGGAGCACACAAGAGCAGC
		R: TTGTGGAACAGCTTCAGCCA
*β-Actin*	Cs1g05000	F: CCGACCGTATGAGCAAGGAAA
		R: TTCCTGTGGACAATGGATGGA

Gene IDs are from the genome database of *Citrus sinensis*.

## Data Availability

All of the data supporting the findings of this study are included in this article.
